# Traumatic Ruptured Hepatic Cyst Managed by Laparoscopic Fenestration Assisted With Indocyanine Green (ICG) Fluorescence: A Case Report

**DOI:** 10.1155/cris/2283895

**Published:** 2026-09-29

**Authors:** Rikuto Uryu, Hirofumi Hirao, Kazuyuki Nagai, Satoshi Ogiso, Shinya Okumura, Tomoaki Yoh, Kei Yamane, Etsuro Hatano

**Affiliations:** ^1^ Division of Hepato-Pancreato-Biliary Surgery and Transplantation, Department of Surgery, Kyoto University Graduate School of Medicine, Kyoto, Japan, kyoto-u.ac.jp

**Keywords:** hepatic cyst, indocyanine green, laparoscopic fenestration, traumatic rupture

## Abstract

Nonparasitic hepatic cysts are common and generally asymptomatic. However, complications such as infection, hemorrhage, or rupture of the cyst necessitate therapeutic intervention. We report a rare case of traumatic rupture of a large hepatic cyst managed laparoscopically with the assistance of indocyanine green (ICG) fluorescence. A 57‐year‐old woman had been followed for 2 years for a large cystic lesion in segments 4 (S4) and 8 (S8) of the liver, suspected to be a mucinous cystic neoplasm (MCN). As the S4 cyst grew to 15 cm, surgical resection was planned. However, she presented to our emergency department after blunt abdominal trauma sustained from a fall at home. Computed tomography (CT) revealed rupture of the S4 cyst with massive ascites. Although initially managed conservatively with little abdominal symptoms, she developed worsening abdominal pain and progressive anemia 2 days postadmission, raising concern for cyst‐related hemorrhage and possible bile leakage. Emergent laparoscopic surgery revealed hemoperitoneum and bleeding from the ruptured cyst wall. ICG was administered intravenously immediately after anesthesia induction to visualize the communication between the cyst and the biliary tract by fluorescence imaging. Then, laparoscopic fenestration was performed under ICG guidance. The patient was discharged on postoperative day (POD) 6 without any complications. We successfully treated a rare case of traumatic hepatic cyst rupture by laparoscopic fenestration with ICG imaging. ICG fluorescence was a helpful adjunct for intraoperative assessment of the potential bile duct in the cyst, ensuring the safety of the operative procedure.

**Summary**

Traumatic rupture of a hepatic cyst is a rare condition; however, bile leakage from the cyst wall can occasionally pose a clinical challenge. In this case, indocyanine green (ICG) fluorescence imaging enabled clear differentiation of the bile ducts and hepatic parenchyma from the cyst wall, allowing laparoscopic surgery to be performed safely.

## 1. Introduction

Nonparasitic hepatic cysts are relatively common benign liver lesions found in ~1%–5% of the general population. They are reported to be more prevalent in women and often discovered incidentally with recent advances in diagnostic imaging [1]. They are asymptomatic in most cases, and therapeutic intervention is considered when they become symptomatic (e.g., abdominal pain or fullness) or when complications such as infection, hemorrhage, compressive symptoms (e.g., jaundice), or rupture are suspected [1, 2].

The etiologies of hepatic cyst rupture include spontaneous rupture [3, 4], infection [5], medication [6], and trauma [1, 2]. The rupture of a hepatic cyst is a rare complication but requires prompt diagnosis and urgent treatment since it can cause hemorrhagic shock [7] or biliary peritonitis [8]. The rupture of a hepatic cyst may result in biliary peritonitis when the cyst communicates with the intrahepatic biliary system. Trauma can disrupt adjacent bile ducts, allowing bile to leak into the peritoneal cavity, leading to chemical peritonitis. Hence, the strategy to prevent bile leakage in the treatment of traumatic rupture is warranted.

Recent advances in surgical imaging technologies have facilitated the widespread adoption of indocyanine green (ICG) fluorescence imaging, enabling real‐time visualization of anatomical structures otherwise invisible to the naked eye. This technique has since been extended to various surgical disciplines, not only in hepatobiliary surgery, with expanding indications that enhance intraoperative decision‐making and surgical safety [9].

Herein, we report a case of traumatic rupture of a large nonparasitic hepatic cyst, which was successfully treated with emergent laparoscopic fenestration using ICG fluorescence imaging to minimize perioperative complications.

## 2. Case Presentation

A 57‐year‐old woman was referred to our hospital 2 years earlier after a large hepatic cystic lesion was incidentally identified during an evaluation for abdominal fullness. Her medical history was unremarkable except for radial nerve palsy. Contrast‐enhanced computed tomography (CT) demonstrated a large cystic lesion involving hepatic segments 4 (S4) and 8 (S8) (Figure 1A). However, CT showed no evidence of renal cysts, making autosomal dominant polycystic kidney disease (ADPKD) an unlikely cause of the multiple hepatic cysts. Furthermore, the patient’s medical and family history did not suggest any hereditary disorder known to be associated with multiple hepatic cysts. Magnetic resonance imaging (MRI) revealed partial enhancement and internal solid components within the cyst, in which the possibility of a mucinous cystic neoplasm (MCN) could not be completely ruled out but whose contents were most likely organized hemorrhage (Figure 1B). The patient was therefore managed with careful radiologic surveillance. During follow‐up, the cystic lesion in S4 gradually enlarged to a maximum diameter of 15 cm, prompting elective surgical resection.

**Figure 1:**
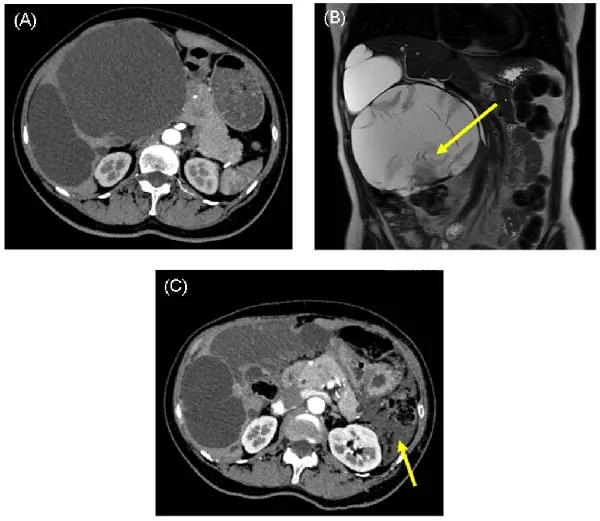
CT and MRI imaging findings of a hepatic cyst. (A) Pretrauma CT scan. (B) T1‐weighted MRI (the arrow indicates a suspected organized hematoma). (C) Postinjury CT showing collapse of the cyst with associated ascites (arrow).

However, before the scheduled operation, the patient sustained blunt abdominal trauma following a fall at home and presented to the emergency department with abdominal discomfort. Abdominal CT revealed the rupture of the S4 cyst, accompanied by intraabdominal fluid consistent with ascites (Figure 1C). Given her stable vital signs, mild abdominal symptoms, and no progression of anemia assessed by a blood test, conservative management was initially pursued. 2 days after admission, the patient developed worsening abdominal pain, and laboratory studies demonstrated progressive anemia and an elevation of C‐reactive protein, an indicator of an inflammatory response (Table 1). In light of suspected ongoing hemorrhage and possible bile leakage from the ruptured cyst, emergent laparoscopic surgery was indicated. The surgery was performed with the patient in the supine position under general anesthesia. Immediately after the induction of anesthesia, 2.5 mg of ICG was administered intravenously. A 12‐mm trocar was inserted into the umbilicus, and pneumoperitoneum was established. Laparoscopic exploration revealed bloody ascites in the right paracolic gutter, bilateral subphrenic spaces, and the pouch of Douglas, confirming hemorrhagic rupture of the hepatic cyst (Figure 2A,B). Additional ports were placed, including 5‐mm trocars in both flanks and a 12‐mm trocar between the umbilical port and the right flank ports.

**Figure 2:**
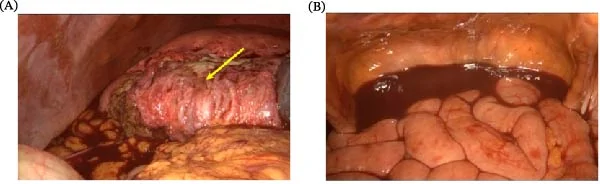
Intraoperative laparoscopic view. (A) A ruptured cyst (arrow) and surrounding hemorrhagic ascites. (B) Bloody ascites in the Douglas pouch.

**Table 1:** Preoperative laboratory data.

	Before	Posttrauma	2 days after admission	Unit
WBC	4.68	10.84	6.71	10^9^/L
Hb	15.1	14.5	12.9	g/dL
Plt	253	256	213	10^9^/L
AST	29	29	25	U/L
ALT	13	14	8	U/L
γ‐GTP	50	119	87	U/L
T‐Bil	0.9	0.6	1.2	mg/dL
D‐Bil	0.1	0.1	0.3	mg/dL
CRP	0.16	0.19	15.03	mg/dL

Abbreviations: γ‐GTP, gamma‐glutamyltransferase; ALT, alanine aminotransferase; AST, aspartate aminotransferase; Bil, bilirubin; CRP, C‐reactive protein; Hb, hemoglobin; Plt, platelet; WBC, white blood cell.

Approximately 120 mL of bloody ascites was aspirated, revealing a defect in the cyst wall of S4 with oozing hemorrhage, which was identified as the bleeding source (Figure 3A). The cyst cavity contained an old, chocolate‐colored hematoma, which was thoroughly irrigated and aspirated. After aspirating the cyst contents, we carefully inspected the inner surface of the cyst wall and found no gross evidence of a neoplastic lesion. Based on the preoperative imaging findings and the intraoperative appearance, we diagnosed the lesion as a simple hepatic cyst with organized hemorrhage and therefore proceeded with laparoscopic fenestration. Thereafter, using an ICG fluorescence imaging mode (1688 AIM 4K; Stryker), the liver surface and cyst cavity were carefully inspected. We identified the boundary of ICG fluorescence as the interface between the hepatic parenchyma and the cyst (Figure 3B). No fluorescence suggestive of bile leakage was observed from the rupture site or the cutting edge, indicating the absence of significant communication with the biliary tree, although an ICG‐positive area was observed on the inner surface of the cyst wall, which was considered to be derived from the underlying hepatic parenchyma (Figure 3C). The cyst wall, including the bleeding point, was resected and fenestrated (deroofed) using a vessel‐sealing device (LigaSure; Medtronic). The left subphrenic space, the pouch of Douglas, and the right subphrenic space were irrigated with saline. The cyst wall was collected in an E.Z. Purse retrieval bag. Hemostaticc powder (Arista) was applied to the cut edge of the cyst wall. A 19‐Fr round J‐Vac drain was placed from the 5‐mm port site on the right flank, and the incisions were closed to complete the operation. The detailed timeline of the intraoperative procedure is as follows: the start of surgery was defined as time 0. Intravenous administration of 2.5 mg of ICG was performed 38 min before the start of surgery. Pneumoperitoneum was established, and laparoscopic exploration was initiated at 2 min. Bloody ascites and a ruptured hepatic cyst were identified at this stage. The initial fluorescence assessment was performed at 5 min, and cyst fenestration was initiated at 22 min. At 60 min, the cyst‐wall specimen was retrieved, followed by the final fluorescence assessment. The pneumoperitoneum was released at 61 min, and the operation was completed at 71 min. The resected specimen measured 10.0 × 3.0 × 0.5 cm. Histological examination revealed a hyalinized, thickened cyst wall lined by a single layer of cuboidal to flattened epithelium, with associated fibrin and hemosiderin deposition. These findings were consistent with the diagnosis of a simple liver cyst. The postoperative course was uneventful, with no complications. Since the cyst wall was not completely resected, the possibility of an underlying neoplastic lesion cannot be entirely excluded. Hence, the patient has been undergoing regular radiological follow‐up with CT and MRI to monitor for recurrence or the development of neoplastic changes. CT performed 3 months after discharge confirmed successful fenestration of the cyst, with no evidence of hemorrhage or neoplastic lesions. A follow‐up CT obtained 9 months later demonstrated no enlargement of the cyst or newly developed neoplastic lesions. In addition, MRI performed 15 months postoperatively revealed no evidence of tumor formation (Figure 4). As multiple hepatic cysts remain in other segments of the liver, the patient will continue to undergo regular surveillance with CT and/or MRI to monitor for recurrence or the development of neoplastic changes.

**Figure 3:**
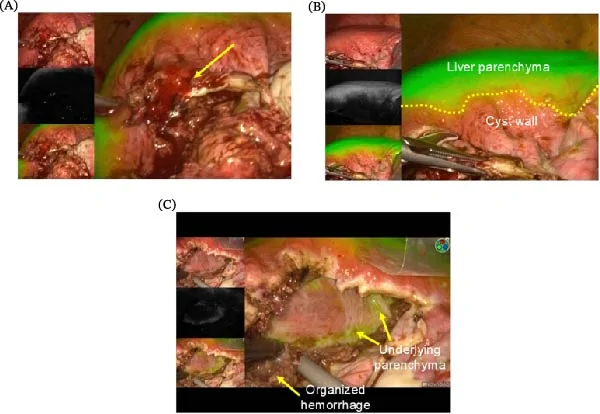
Findings of intraoperative ICG fluorescence imaging. (A) Bleeding site from the cyst wall (arrow). (B) The interface between the cyst wall and the hepatic parenchyma (dotted line). (C) No fluorescence suggestive of bile leakage from the rupture site or the cutting edge.

**Figure 4:**
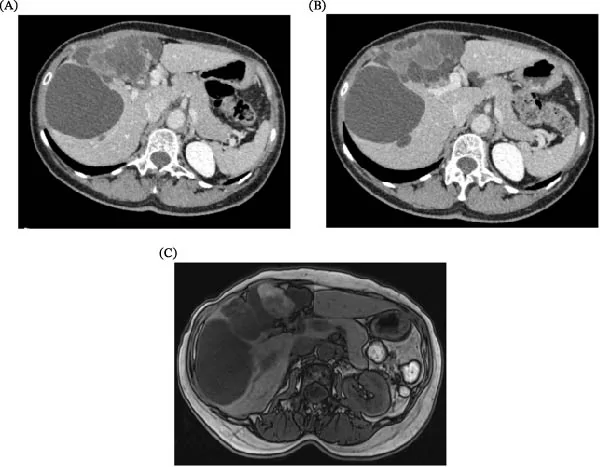
Postoperative follow‐up imaging at 3 (A), 9 (B), and 15 months (C).

## 3. Discussion

The rupture of a hepatic cyst is a rare event but may require prompt intervention. A review of the literature from 1999 to 2025 identified 25 cases of hepatic cyst rupture with detailed descriptions, as summarized in Table S1. A literature search was performed using PubMed to identify previously reported cases of ruptured noninfectious and nonneoplastic hepatic cysts. The final search was conducted on January 22, 2026. The following search terms were used in various combinations: “hepatic cyst,” “simple hepatic cyst,” “nonparasitic hepatic cyst,” “rupture,” “spontaneous rupture,” and “traumatic rupture.” The inclusion criteria were as follows: (1) case reports or case series describing spontaneous or traumatic rupture of simple hepatic cysts, (2) cysts confirmed to be noninfectious and nonneoplastic, and (3) articles published between 1999 and 2025. The exclusion criteria included (1) hydatid or parasitic cysts, (2) infected hepatic cysts or liver abscesses, (3) polycystic liver disease associated with ADPKD, and (4) cystic neoplasms such as MCN. The cases of ruptured hepatic cysts are more commonly observed in relatively elderly patients (mean age, 65 years) and predominantly in women (60%). The mean cyst size was 12.8 cm, and about 60% of cysts were located in the right hepatic lobe. While 84% of ruptures are spontaneous, only 8% (including our case) are traumatic. Clinically, 92% of cases present with ascites, often hemorrhagic, which aligns with our findings. In the treatment of a ruptured liver cyst, the control of hemorrhage is essential. However, the communication between the cyst wall and the biliary system can cause bile leakage and subsequent biliary peritonitis, which represents another primary clinical concern.

ICG is a water‐soluble tricarbocyanine dye that binds rapidly to plasma proteins after intravenous administration. ICG is selectively taken up by hepatocytes via active transport mechanisms and then excreted unchanged into the bile. Following biliary excretion, ICG accumulates transiently within the bile ducts. When the operative field is illuminated with near‐infrared (NIR) light (wavelength ~750–810 nm), ICG emits fluorescence at a slightly longer wavelength (around 830 nm) [10]. This fluorescence can be detected by NIR imaging systems, allowing for real‐time visualization of the biliary anatomy, including the extrahepatic and intrahepatic bile ducts. A randomized controlled trial (RCT) in the cohort of open liver resection demonstrated that the operation with ICG fluorescence imaging significantly reduced postoperative bile leakage (10% vs. 0%), proving the efficacy of the “find and seal” targeted strategy [11]. Additionally, a recent report suggests that ICG fluorescence cholangiography significantly improves the success rate of common bile duct identification in laparoscopic cholecystectomy [12]. Thus, ICG fluorescence imaging has become a valuable tool for visualizing the biliary anatomy in hepatobiliary surgery and has contributed to reducing biliary complications.

In the context of treatment for rupture, conservative therapy (16%) and percutaneous/IVR interventions (12%) are options for patients with a stable general condition, while surgical intervention is often selected (72%; open: 36% and laparoscopic: 36%). Laparoscopic fenestration (deroofing) has emerged as a minimally invasive strategy for the treatment of hepatic cysts, even in cases of rupture, as first reported in 2005, although open surgery may be required in cases of hemodynamic instability [7]. A key challenge in the laparoscopic approach is the assessment of potential biliary communication between the liver parenchyma and cystic lesion. To overcome this issue, there have been increasing reports of hepatobiliary surgery using ICG fluorescence imaging during laparoscopic procedures [13, 14].

The optimal timing and dose of ICG administration for cholangiography are debatable. Although the optimal timing and dosage of ICG administration vary depending on the disease and surgical context, a consensus has emerged from multiple studies [13]. For example, in laparoscopic cholecystectomy, 2.5 mg of ICG is typically administered 30–60 min before surgery [15]. For tumor imaging, ICG is administered at a dose of 0.5 mg/kg ~10–14 days before surgery. In anatomical hepatectomy, negative staining is achieved by administering 2.5 mg of ICG after ligation of the Glissonean pedicle supplying the tumor, whereas for positive staining, 0.125–0.25 mg of ICG is injected into the portal vein branch supplying the resection area. On the contrary, the optimal timing and dosage of ICG administration during laparoscopic surgery for ruptured hepatic cysts remain largely unknown. In our case, we administered 2.5 mg of ICG intravenously immediately after anesthesia induction to confirm communication between the rupture site and the biliary tract, delineate the boundary between the hepatic parenchyma and the cyst wall, and finally assess bile leakage from the cut edge after fenestration. Indeed, we obtained a sufficient signal, confirming that no ICG fluorescence was observed at the rupture site, indicating no obvious communication with the biliary tract, thereby allowing us to make the intraoperative decision to avoid a major hepatectomy and proceed with cyst fenestration. Therefore, we assume that in an emergency setting like traumatic cyst rupture, where preoperative administration is difficult, administration at the time of anesthesia induction may be a feasible option for assessing active bile leakage. However, the optimal dosage and timing of ICG for the management of ruptured hepatic cysts should be discussed on a case‐by‐case basis; further cases are warranted. We recognize that ICG has several limitations. For example, ICG fluorescence can be detected only to a depth of ~5–10 mm. In addition, the fluorescence signal may be attenuated in the presence of inflammation, scar tissue, or obesity, and ICG is contraindicated in patients with an iodine allergy [16]. Therefore, the absence of ICG fluorescence alone cannot completely exclude cystobiliary communication. Accordingly, intraoperative findings should be assessed comprehensively, including direct inspection of the cyst cavity and cut surface, measurement of bilirubin levels in the cyst fluid, and, when feasible, intraoperative cholangiography. In the present case, neither intraoperative cholangiography nor biochemical analysis of the cyst fluid was performed. However, the postoperative drain fluid on postoperative day (POD) 1 showed a total bilirubin level of 5.5 mg/dL and a direct bilirubin level of 0.4 mg/dL. Given the low proportion of direct bilirubin and the serosanguineous appearance of the drainage fluid, these findings were considered more consistent with blood contamination than with bile leakage. Ultimately, no clinical evidence of postoperative bile leakage was observed. Nevertheless, even in disease conditions that require emergency surgery other than our present case, the use of ICG fluorescence‐guided surgery is actively recommended to improve surgical outcomes [9], and its potential applications are expected to continue expanding.

In conclusion, we experienced a rare case of traumatic hepatic cyst rupture safely managed by laparoscopic cyst fenestration. The adjunctive use of ICG fluorescence, even when administered in an emergency setting, helped assess the bile leakage risk and facilitate a safe, minimally invasive surgical approach.

## Author Contributions

**Rikuto Uryu**: data curation, formal analysis, writing – original draft. **Hirofumi Hirao**: conceptualization, funding acquisition, supervision, validation, writing – review and editing. **Kazuyuki Nagai**: formal analysis, investigation, methodology, validation. **Satoshi Ogiso**: methodology, supervision. **Shinya Okumura**: data curation, investigation, validation. **Tomoaki Yoh**: conceptualization, methodology, validation. **Kei Yamane**: formal analysis, methodology, validation. **Etsuro Hatano**: project administration, supervision.

## Funding

This work was supported by the Grant‐in‐Aid for Early‐Career Scientists (Grant 24K19318) from the Ministry of Education, Culture, Science and Sports, Japan.

## Disclosure

All authors read and approved the final manuscript.

## Ethics Statement

This study complied with the Declaration of Helsinki, and patient anonymity was maintained. According to the institutional guidelines, ethical approval was not required for this type of case report.

## Consent

Written informed consent was obtained from the patient for the publication of this manuscript.

## Conflicts of Interest

The authors declare no conflicts of interest.

## Supporting Information

Additional supporting information can be found online in the Supporting Information section.

## Supporting information


**Supporting Information** Table S1: Reported cases of traumatic rupture of hepatic cysts.

## Data Availability

The data supporting the findings of this study are available from the corresponding author upon reasonable request.
